# Oxidative Stress Alters miRNA and Gene Expression Profiles in Villous First Trimester Trophoblasts

**DOI:** 10.1155/2015/257090

**Published:** 2015-08-03

**Authors:** Courtney E. Cross, Mai F. Tolba, Catherine M. Rondelli, Meixiang Xu, Sherif Z. Abdel-Rahman

**Affiliations:** ^1^Department of Obstetrics and Gynecology, The University of Texas Medical Branch, Galveston, TX 77555-1066, USA; ^2^Department of Pharmacology and Toxicology, Faculty of Pharmacy, Ain Shams University, Cairo 11566, Egypt

## Abstract

The relationship between oxidative stress and miRNA changes in placenta as a potential mechanism involved in preeclampsia (PE) is not fully elucidated. We investigated the impact of oxidative stress on miRNAs and mRNA expression profiles of genes associated with PE in villous 3A first trimester trophoblast cells exposed to H_2_O_2_ at 12 different concentrations (0-1 mM) for 0.5, 4, 24, and 48 h. Cytotoxicity, determined using the SRB assay, was used to calculate the IC_50_ of H_2_O_2_. RNA was extracted after 4 h exposure to H_2_O_2_ for miRNA and gene expression profiling. H_2_O_2_ exerted a concentration- and time-dependent cytotoxicity on 3A trophoblast cells. Short-term exposure of 3A cells to low concentration of H_2_O_2_ (5% of IC_50_) significantly altered miRNA profile as evidenced by significant changes in 195 out of 595 evaluable miRNAs. Tool for annotations of microRNAs (TAM) analysis indicated that these altered miRNAs fall into 43 clusters and 34 families, with 41 functions identified. Exposure to H_2_O_2_ altered mRNA expression of 22 out of 84 key genes involved in dysregulation of placental development. In conclusion, short-term exposure of villous first trimester trophoblasts to low concentrations of H_2_O_2_ significantly alters miRNA profile and expression of genes implicated in placental development.

## 1. Introduction

Preeclampsia (PE), which affects 3% to 8% of pregnant women, remains a major cause of short- and long-term maternal and neonatal morbidity and mortality. It is a medical condition characterized by* de novo* hypertension in pregnancy (diastolic > 90 mm Hg) after 20-week gestation with high proteinuria (>300 mg) [1]. PE is thought to result from a combination of many factors including shallow trophoblast invasion, failed maternal spiral artery remodeling, and an increase in endothelial activation leading to placental hypoxia, reactive oxygen species (ROS) generation, apoptosis and necrosis of trophoblasts, and systemic activation of inflammatory processes in the mother [1]. Superoxide anions generated endogenously or exogenously can rapidly be converted to hydrogen peroxide (H_2_O_2_) [2] and studies showed a significant elevation of H_2_O_2_ levels in the bloodstream of women with PE [3, 4]. H_2_O_2_ levels are also significantly higher in preeclamptic placentas compared to normotensive placentas at term [5]. Moreover, evidence supports an early increase in oxidative stress in the placenta by the end of the first trimester before the clinical development of PE [6–8]. Oxidative stress can induce endothelial dysfunction and vasoconstriction [9]. Therefore, the relationship between oxidative stress and PE is a vicious cycle where increased oxidative stress can induce PE and the occurrence of PE also exacerbates oxidative stress. The age-adjusted incidence of PE in the United States increased almost 25% from 1987 to 2004 [10]. With disease risk on the rise but no effective way to predict its development, it is crucial to understand the early etiologic mechanisms of PE to develop early detection biomarkers and possible preventive measures for the disease.

MicroRNAs (miRNAs) are short 20–22 single strand regulatory RNAs that function by inhibiting translation of their targets or promoting target RNA degradation [11, 12]. There is a temporal and placental-specific pattern of miRNA expression [13]. This pattern includes two large imprinted miRNA clusters, one located at chromosome 19q13.41 (C19MC) and another at 14q32 (C14MC). Expression of C14MC decreases while expression of C19MC increases as pregnancy progresses [14]. Women with PE, eclampsia, and HELLP (Hemolysis, Elevated Liver enzymes, Low Platelets) syndrome have significant alterations in the placental miRNA profile. For example, dysregulation of C19MC expression is seen in preeclamptic placentas [15]. In addition, miR-210, which regulates hypoxia-inducible factor 1, is commonly found to be upregulated in PE women [16]. Dysregulated expression of a number of other miRNAs, such as the oncogenic miR-17 family, occurs in preeclamptic placentas but the temporal change is unknown [17–20]. Given the reported changes in miRNAs observed in PE, early detection, protection, and regulation of miRNAs may help decrease the impact of this disease.

The relationship between oxidative stress and miRNA changes in placenta as a potential mechanism involved in PE is not fully elucidated. In the present study, we investigate the impact of oxidative stress on miRNA and mRNA expression profiles, with specific emphasis on mRNAs of genes known to be associated with PE, in villous first trimester 3A cytotrophoblast cell line.

## 2. Materials and Methods

### 2.1. Chemicals and Reagents

The villous 3A cytotrophoblast first trimester placental cell line (CRL-1584) was purchased from American Type Culture Collection (ATCC) (Manassas, VA). The miRNA and mRNA kits with appropriate real-time reagents and miRNEasy were purchased from Qiagen (Valencia, CA). CytoScan SRB cell cytotoxicity assay kit was purchased from G-biosciences (St. Louis, MO). Eagle's Minimum Essential Medium (EMEM) and 0.25% trypsin were Gibco brand (Life technologies, Grand Island, NY). Fetal bovine serum (FBS) was acquired from Atlanta Biologicals (Lawrenceville, GA). All other chemicals were purchased from Sigma-Aldrich Co. (St. Louis, MO, USA) and were of the highest purity available.

### 2.2. Cell Culture and H_2_O_2_ Treatment

Villous 3A cytotrophoblast cells were maintained in 75 cm^2^ flasks at 37°C/5% CO_2_ in complete medium consisting of EMEM supplemented with 10% fetal bovine serum and 1% penicillin/streptomycin. Cells were passaged at ~90% confluency. H_2_O_2_ exposures were performed in complete medium for the times and concentrations specified in a humidified incubator at 37°C supplemented with 5% CO_2_.

### 2.3. Determination of H_2_O_2_ IC_50_

Cells were seeded in 12-well plates at a density of 0.25 × 10^5^ cells/well and were allowed to attach for 24 hours. Growth media were replaced either with fresh media for mock exposure or with one of 12 different concentrations of H_2_O_2_ (0 to 1 mM) in complete medium for 0.5, 4, 24, or 48 h without further media replacement. Cytotoxicity was assessed at the end of H_2_O_2_ exposure using the SRB assay as previously described [21]. Briefly, after exposure, cells were fixed with 10% tricholoroacetic acid for one hour and then stained with 0.4% SRB dissolved in 1% acetic acid. Excess dye was washed and wells were air-dried. Bound dye was solubilized in 10 mM Tris (pH 7.4) and absorbance was measured at 560 nm using a microplate reader (TECAN GENios Pro, Männedorf, Switzerland). Results were expressed as the relative percentage of absorbance compared to control. All experiments were performed in triplicate. Half-maximal inhibitory concentration (IC_50_) was calculated using SigmaPlot, version 12.3 (Systat Software Inc., San Jose, CA, USA).

### 2.4. Assessment of Oxidative Stress Markers

Reduced glutathione (GSH) levels and superoxide dismutase (SOD) activity were assessed in the cell lysates as markers for oxidative stress [22, 23]. To determine the level of GSH, an aliquot (0.2 mL) of cell lysate was added to a tube containing 1.7 mL phosphate buffer and 0.1 mL Ellman's reagent; then the absorbance was read at 412 nm within 5 min [24]. The results were expressed as mg/*μ*g protein. SOD activity was assessed in the cell lysates using an assay that relies on the ability of SOD enzyme to inhibit the phenazine methosulphate- (PMS-) mediated reduction of nitroblue tetrazolium dye. The change in absorbance over 5 min was measured at 560 nm [25]. SOD activity was expressed as U/mg protein. Protein levels were determined using the BCA protein assay (Pierce Biotechnology, Rockford, IL, USA). Student's *t*-test was used for statistical comparisons and significant differences were established at *P* < 0.05.

### 2.5. Determination of Effects of H_2_O_2_ Exposure on miRNA and mRNA Expression Profiles

Cells were grown to 90% confluency in 75 cm^2^ flasks and exposed to 25 *μ*M H_2_O_2_ (equivalent to 5% of the IC_50_) in complete medium for 4 h. Total RNA was isolated using the Qiagen miRNEasy Mini Kit and quality/quantity was measured in the Molecular Genomics Core at UTMB. RNA was quantitated spectrophotometrically using a NanoDrop ND-1000 (NanoDrop Technologies, DE). Quality of the purified RNA was assessed by visualization of 18S and 28S RNA bands using an Agilent Bioanalyzer 2100 (Agilent Technologies, CA). Resulting electropherograms were used in the calculation of the 28S/18S ratio and the RNA Integrity Number. Reverse transcription was carried out using either the miScript II RT kit or RT^2^ First Strand and subsequent SYBR green based real-time PCR on a Bio-Rad Chromo4 Real-Time PCR Detector per the manufacturer's recommendation. The miRNA profile screening was performed using miScript Human miRNome PCR Array (MIHS-3216Z, Qiagen, Valencia, CA). RT² Profiler Human Preeclampsia PCR Array (PAHS-163Z, Qiagen, Valencia, CA) was used for gene expression profiling. Data was analyzed using the ΔΔCT method with either the miScript miRNA PCR Array Data Analysis version 3.5 or with the RT^2^ Profiler PCR Array Data Analysis (SABiosciences, Valencia, CA).

## 3. Results

### 3.1. H_2_O_2_-Induced Cytotoxicity in Villous 3A Trophoblasts

**Figure 1:**
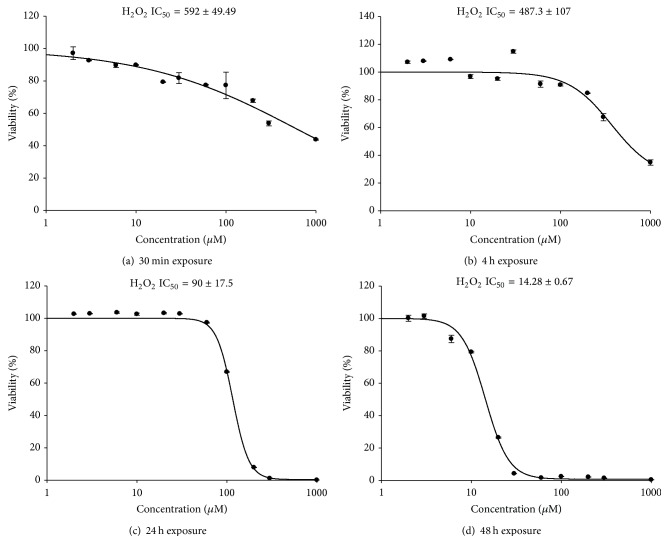
IC_50_ calculation for H_2_O_2_ after 30 min, 4 h, 24 hr, or 48 hr in villous 3A trophoblasts: 3A cells were exposed to varying concentrations (0, 2, 3, 6, 10, 20, 30, 60, 100, 200, 300, and 1000 *μ*M) of H_2_O_2_ and IC_50_ determined using the SRB assay. Data represent *n* = 3 for each time point and concentration. IC_50_ was calculated using SigmaPlot v12.3.

In order to determine the appropriate H_2_O_2_ concentration for further experiments, cells were exposed to varying micromolar concentrations of H_2_O_2_ in complete medium for up to 48 h and cytotoxicity was determined using the SRB assay. Exposure of 3A placental cells to H_2_O_2_ resulted in a time- and concentration-dependent cytotoxic effect. The IC_50_ values for H_2_O_2_ were 592, 487, 90, and 15 *μ*M after 30 min, 4, 24, and 48 h exposures, respectively (Figure 1). A concentration equivalent to 5% of the IC_50_ concentration was used for the miRNAs and mRNA expression profiling experiments. The 4-hour exposure was selected for short-term exposure studies of the effect of H_2_O_2_ on miRNA and mRNA expression profile. The levels of GSH as well as SOD activity were assessed after exposure of the cells to 25 *μ*M H_2_O_2_ for 4 h as markers for oxidative stress status [22, 23]. GSH level in H_2_O_2_ challenged cells was significantly reduced by 30% (from 361.44 ± 10.01 mg/mg protein in untreated cells to 251.35 ± 39.23 in the treated cells; *P* < 0.01). Similarly, SOD activity in cells exposed to H_2_O_2_ was significantly reduced by over 35% (208.9 ± 5.12 U/mg protein in treated cells compared to 321.71 ± 6.78 in the untreated cells; *P* < 0.01).

### 3.2. H_2_O_2_ Alters Normal miRNA Expression Profile in Villous 3A Trophoblasts

**Figure 2:**
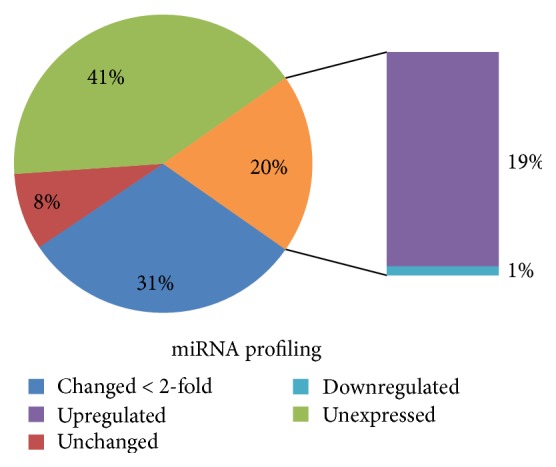
Pie chart representation for miRNome array results after 4 h exposure to H_2_O_2_ in villous 3A trophoblasts.

To investigate the potential effect of oxidative stress on miRNA expression profile in villous 3A trophoblasts, cells were exposed to 25 *μ*M H_2_O_2_ for 4 h in complete medium and total RNA (including miRNA) was isolated using the Qiagen miRNeasy Mini Kit. The Qiagen v16 miRNA Array was used to evaluate the expression of 1008 miRNAs after H_2_O_2_ exposure compared to unexposed control. In our study, 417 miRNAs were not expressed (unevaluable) in the tested cell line. Out of the 591 evaluable miRNAs, 195 were up- or downregulated by at least twofold after H_2_O_2_ exposure (Supplemental Table 1 available online at  http://dx.doi.org/10.1155/2015/257090). The majority of altered miRNAs (95.5%) were upregulated by at least 2-fold and only 4% were downregulated by at least 2-fold (Figure 2). Mir-21, -770, and -596 were downregulated by more than 5-fold, while mir-3907 was downregulated by more than 50-fold. Mir-637, mir-1911, mir-26b, mir-615, let-7a, and let-7f were upregulated more than 50-fold (Supplemental Table 1).

Using TAM (tool for annotations of microRNAs, version 2; http://202.38.126.151/hmdd/tools/tam.html/) analysis, our data indicate that the altered miRNAs fall into 43 clusters and 34 families, with 41 functions identified. Three clusters of miRNAs were significantly altered (*P* < 0.05) after 4 h exposure to 25 *μ*M H_2_O_2_ including hsa-let-7e, -let-106b, and -let-23b clusters (Table 1). Eight miRNA families (let-7, mir-15, mir-17, mir-181, mir-29, mir-329, mir-368, and mir-99) were significantly altered (*P* < 0.05) after H_2_O_2_ challenge (Table 1). Significant alterations (*P* < 0.05) occurred after exposure to H_2_O_2_ in miRNAs involved in critical cellular functions such as angiogenesis, apoptosis, cell proliferation, epithelial-mesenchymal transition, folliculogenesis, granulopoiesis, hormone regulation, human embryonic stem cell regulation, immune response, inflammation, anticell proliferation as well as miRNA tumor suppressors, and onco-miRNAs which can affect cell proliferation and invasion (Table 2). Notably, 21 out of 30 (70%) of evaluable miRNAs of the maternally imprinted miRNA cluster on chromosome 14 (C14MC) that is predominantly expressed in placenta and developing embryonic tissues were altered. Expression of the paternally imprinted chromosome 19 cluster (C19MC) was too low to be evaluated in this study.

### 3.3. H_2_O_2_ Alters mRNA Expression in Villous 3A Trophoblasts

**Figure 3:**
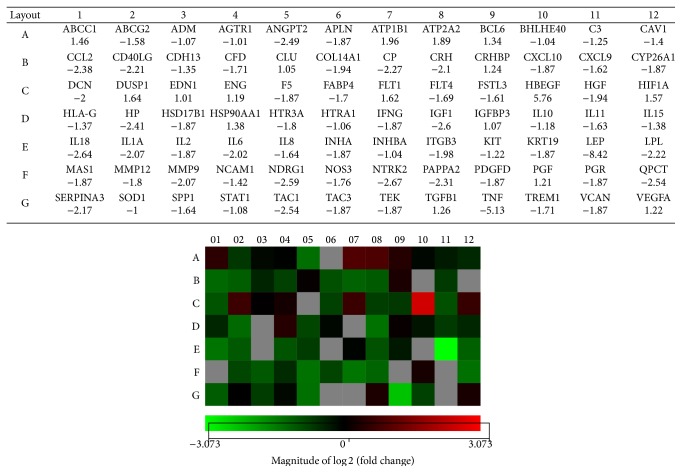
Heat map representation for the differential expression of genes associated with PE after 4 h exposure to H_2_O_2_ in villous 3A trophoblasts. Gene expression is represented in the heat map in the color scale of −3.073–3.073 in green-red color scheme (*n* = 2). Genes evaluated and their locations on the heat map are depicted in the associated table above the heat map.

In order to investigate the effect of oxidative stress on mRNA expression profile, we determined mRNA expression levels of 84 genes potentially involved in preeclamptic pregnancies. Villous 3A cells were mock-exposed in complete medium or exposed to 25 *μ*M H_2_O_2_ in complete medium for 4 hours and total RNA was isolated using the Qiagen miRNeasy Mini Kit. The Qiagen RT^2^ Preeclampsia Array was used to evaluate the expression of these 84 genes after H_2_O_2_ exposure. As shown in Figure 3, of these 84 genes, 22 were up- or downregulated by at least twofold after H_2_O_2_ exposure. Of these, only the HBEGF gene was found to be upregulated while the other 21 genes were downregulated (Table 3).

We used the Qiagen online RT^2^ Profiler PCR Array Data Analysis v3.5 to determine the correlation between the altered miRNAs and mRNAs in our study. Our analysis indicates that 53 of the overexpressed miRNAs in our study putatively target 11 of the downregulated genes (Table 4).

## 4. Discussion

In the current study, H_2_O_2_ exerted a cytotoxic effect in 3A trophoblasts in a concentration- and time-dependent manner. These data are consistent with previous reports [5, 26]. However, the median inhibitory concentration (IC_50_) of H_2_O_2_ in our study after 48 h exposure was 15 *μ*M which is 10 times lower than the IC_50_ reported by Zhou et al. using HTR-8/SVneo cells [5]. In a separate study, Moll et al. [26] used H_2_O_2_ concentrations up to 1000 *μ*M, which is 66 times higher than our IC_50_ concentration, to evaluate apoptosis and proliferation in human term placentas. Murata et al. [27] used a concentration of 100 *μ*M H_2_O_2_ to determine apoptotic and invasion rates in term extravillous trophoblasts at 24 hours. This concentration is comparable to our IC_50_ of 90 *μ*M at 24 hours. This variability in results can be attributed to the variation in sensitivity of the cells studied to determine H_2_O_2_ toxic effects. The villous trophoblast cell line 3A used in our study, which is a first trimester placental cell line, seems to be more sensitive to H_2_O_2_ damage compared to HTR-8/SVneo cells or term primary cells used in these investigations [5, 26]. Our studies were carried out using 5% of the IC_50_ of H_2_O_2_ (25 *μ*M at 4 h) which is a subcytotoxic concentration. This level of exposure was chosen to mimic H_2_O_2_ concentration in PE placentas previously reported by Zhou et al. [5]. Given the reported duplicitous nature of ROS depending on their levels [28], we evaluated the oxidative stress status of the cells at this exposure level by assessing GSH level and SOD activity in the challenged cells as markers of oxidative stress [22, 23]. Both GSH and SOD are of major importance in intracellular redox regulation [29, 30]. Our results revealed a significant reduction in GSH level and SOD activity indicating oxidative stress at this exposure level.

Reports indicate that miRNAs regulate migration, invasion, apoptosis, and proliferation of trophoblasts as well as angiogenesis within the placenta, although the functions of only a few miRNAs have been characterized [18]. Expression changes of miRNAs that regulate these functions have been reported in PE. Wang et al. [20] demonstrated an increase in miR-17, -20a, and -20b in PE. These miRNAs target the angiogenesis factors EPHB4 and ephrin-B2 and could be responsible for the decreases in angiogenesis seen in PE [20]. Upregulation of miR-29b results in increased apoptosis with a corresponding decrease in invasion and angiogenesis in trophoblast cells [31]. MiR-155 targets cyclin D1 and the angiogenic regulating factor CYR61; both are downregulated in PE [32, 33]. Overexpression of miR-210 in extravillous trophoblasts in culture results in decreased cell invasion [34]. MiR-376c, which is lower in both placenta and serum of preeclamptic women, reduces protein levels of ALK5 and ALK7, thus increasing cell proliferation [35].

In our study, after 4 h of H_2_O_2_ exposure, expression of 195 of evaluable miRNAs (CT < 35) was altered by at least twofold. Of these, only seven were downregulated. We chose to focus on the short-term exposure to H_2_O_2_ for the determination of miRNA profile as other studies show a rapid (<4 h) miRNA expression response and subsequent downregulation of protein targets [36]. Our data are in partial agreement with published studies that examined miRNA changes in PE, with some conflicts. There were 6 miRNAs reported to be either up- or downregulated in PE that were not altered in our system. These include miR-210 [37], -34a [38], -149 [19], -19b [39], -92b, and -197 [17]. Two miRNAs, miR-194 and 195, were upregulated in our system in contrast to reported downregulation in the literature [19, 40]. The discrepancy in results is not surprising since most studies were conducted on term placental tissue while we used a first trimester cytotrophoblast cell line. Expressions of placental miRNAs are known to be cell- and trimester-specific [14, 41]. In addition, PE often occurs concurrently with IUGR (intrauterine grown restriction) and preterm labor, both of which have their own miRNA pattern which can thus produce conflicting data. The placental mammal specific miRNA cluster on chromosome 14 (C14MC) predominantly expressed in placenta and embryonic tissues and maternally imprinted had 21 out of 30 (70%) of evaluable miRNAs altered in our study. Meanwhile, expression of the paternally imprinted C19MC cluster was not detected. This expression pattern is in agreement with that found by Morales-Prieto et al. [14], showing higher expression of the C14 cluster in the first trimester with very little expression of C19 miRNAs. Although there are some data indicating an increase in C19MC miRNAs in PE, this increase is most likely due to a loss of methylation as this miRNA cluster is paternally imprinted [15]. Evidence suggests that hypomethylation of multiple genes contributes to early onset PE [42]. Data is mostly lacking for the role of the C14MC cluster in PE. MiR-483-5p was not altered while miR-377 was significantly upregulated in one study of preeclamptic women [16]. Yet, neither of these miRNAs could be evaluated in our study due to the high cycle number in our assay (CT > 35).

The expression profile of 84 genes known or suspected to be altered in PE was examined in this study (Figure 3). These genes are involved in different pathways including pregnancy maintenance, oxidative stress, hormones, growth factors, endothelial cell signaling, and signal transduction. As shown in Table 3, of these 84 genes, 22 were up- or downregulated by at least twofold after H_2_O_2_ exposure. While it is difficult to provide mechanistic explanations to all changes observed in the expression profiles of the 22 genes, it is possible to provide some explanations based on the putative function of these genes in PE and through the correlation of our miRNA and mRNA data. For example, expression of Heparin-binding EGF-like growth factor (HBEGF) was upregulated by 5.8-fold in cells challenged with H_2_O_2_. HBEGF is expressed in both villous and extravillous trophoblasts through the first trimester [43] and is known to act as a survival factor that hampers apoptosis [44] triggered by oxidative stress or other factors [26, 45]. Therefore, its induction under the current experimental conditions could be a cellular defense against oxidative stress known to be associated with PE.

Our data showed that the expression of monocyte chemoattractant protein-1 (CCL2) was downregulated, while the expression of its putative regulator mir-374a-5p was upregulated. CCL2 is known to be expressed in first trimester trophoblasts [46] and is responsible for the activation and recruitment of macrophages to the developing placenta to aid in tissue remodeling after implantation [47]. The expression of CCL2 in first trimester trophoblasts was reported to be regulated by tumor necrosis factor (TNF-*α*) [48]. In our study, expression level of TNF-*α* was also downregulated as was angiopoietin-2 (ANGPT2) when exposed to increased oxidative stress. ANGPT2 is crucial in regulating vascular remodeling through its interaction with endothelial cell Tie-2 receptor [49]. The levels of ANGPT2 were found to be lower in PE compared to normal pregnancy [50]. Similarly, we found the insulin-like growth factor-1 (IGF-1) expression to be decreased. IGF-1 was reported to be decreased in placental tissues from women suffering PE [51]. Of note is that 38 miRNAs that putatively regulate IGF-1 (Table 4) were upregulated in our study. The pregnancy-associated plasma protein A2 (PAPPA2) is an insulin-like growth factor-binding protein (IGFBP) protease expressed at high levels in the placenta. Increased levels of PAPPA2 in PE suggest a compensatory response to abnormal placentation, which might increase insulin-like growth factor (IGF) availability and promote fetoplacental growth [52]. However, PAPPA2 was downregulated in our study, which might also explain the observed reduction in IGF-1 expression in the current work.

The levels of matrix metalloproteinase-9 (MMP9) were decreased in trophoblast cells exposed to H_2_O_2_. MMP9 is an important factor for extracellular matrix remodeling and is responsible for the invasiveness of trophoblasts [53]. Its deficiency induced phenotypic changes that mimic PE in mice [54] and its levels are reduced in pregnancies complicated with PE [55]. Moreover, the level of corticotrophin releasing hormone (CRH) was also downregulated by H_2_O_2_ stress. CRH is released by trophoblasts to promote embryo implantation [56].

Leptin (LEP) gene expression was downregulated in our study. In silico analysis predicted the LEP to be a target of four miRNAs (mir-27b-3p, mir-29a-3p, mir-29c-3p, and mir-9-5p; Table 4). All of those miRNAs were overexpressed under the current experimental conditions. Our findings contrast other reports indicating that LEP is overexpressed in placental tissues at term in PE [57]. It should be noted however that most published data is derived from studies of term trophoblasts or placental tissues while our study focuses on first trimester trophoblasts, which mimics the early stage of pregnancy. This can explain some of the discrepancies in gene expression data observed between our study and others.

Our study has some limitations which we acknowledge. We examined only short-term effects of high H_2_O_2_ exposure on cell viability and short-term effects of low H_2_O_2_ exposure on miRNA and mRNA gene expression profiles. Gene expression changes can differ due to hypoxic versus normal growth conditions or short- versus long-term exposure. This study focuses only on short-term exposure and cytotoxicity due to the reported rapid changes in miRNA expression after initial exposure [36] but will be expanded in the future to include long-term genetic changes. In addition, a change in miRNA or mRNA expression does not necessarily lead to a change in encoded protein levels, protein modification, or protein function. Such information would be generated from specifically designed studies based on our findings. Lastly, we are using an SV40-transformed first trimester cell line rather than primary cells or choriocarcinoma cancer cells, which, to our knowledge, is one of the best available models for studies such as ours. Although the* in vitro* approach provides conformity of cell type over multiple experiments,* in vitro* response to ROS may not totally mimic the* in vivo* response.

In summary, our data indicate that short-term exposure of 3A villous first trimester trophoblasts to H_2_O_2_ significantly alters miRNA profile and mRNA expression of genes implicated in defective placental development. Our data, which indicate that oxidative stress alters miRNAs and RNAs expression, could partially explain some of the early changes in gene expression profiles and miRNA observed in PE.

## Supplementary Material

A complete list of altered miRNAs after 4 hours H_2_O_2_ exposure is available in supplementary table 1.

## Figures and Tables

**Table 1:** Clusters and families of miRNAs significantly altered by H_2_O_2_ in villous 3A trophoblasts.

	Count	Percent^b^	*P* value^c^
Cluster^a^			
hsa-let-7e cluster	3	100	0.0418
hsa-let-106b cluster	3	100	0.0418
hsa-let-23b cluster	3	100	0.0418
Family^a^			
let-7 family	9	100	6.40*e* − 5
mir-15 family	4	100	0.0144
mir-17 family	6	75	0.0237
mir-181 family	4	100	0.0144
mir-29 family	3	100	0.0418
mir-329 family	3	100	0.0418
mir-368 family	3	100	0.0418
mir-99 family	3	100	0.0418

^a^Analysis of overexpressed miRNAs was performed by TAM (tool for annotations of miRNAs, version 2). Size of miRNA category was set as 1–100.

^b^Percent of miRNA changed between treated and controls cells within the cluster or family.

^c^ *P* < 0.05 indicates a significant number of miRNAs altered within a cluster/family.

**Table 2:** Functional pathways regulated by miRNAs altered by H_2_O_2_ in villous 3A trophoblasts.

Function^a^	Count	Percent^b^	*P* value^c^
Angiogenesis	14	61	7.9*e* − 3
Apoptosis	22	56	3.10*e* − 3
Bone regeneration	17	61	3.41*e* − 3
Cell cycle related	29	50	7.50*e* − 3
Cell proliferation	16	62	3.76*e* − 3
Epithelial-mesenchymal transition	24	63	1.72*e* − 4
Folliculogenesis	7	100	5.67*e* − 4
Granulopoiesis	9	90	4.48*e* − 4
Hormones regulation	29	54	1.77*e* − 3
Human embryonic stem cell (hESC) regulation	36	51	1.80*e* − 3
Immune response	29	73	3.36*e* − 7
Inflammation	22	65	2.02*e* − 4
Adipocyte differentiation	16	64	2.08*e* − 3
Anticell proliferation	11	100	1.07*e* − 6
Brain development	12	63	9.45*e* − 3
Cell death	31	62	2.54*e* − 5
Cell division	11	73	2.29*e* − 3
Cell fate determination	12	55	0.0415
Hematopoiesis	18	62	1.77*e* − 3
Lipid metabolism	11	58	0.0304
miRNA tumor suppressors	24	65	9.08*e* − 5
Onco-miRNAs	19	61	1.61*e* − 3

^a^Analysis of altered miRNAs was performed by TAM (tool for annotations of miRNAs, version 2). Size of miRNA category was set as 1–100.

^b^Percent of miRNA changed between treated and controls cells within a pathway/function.

^c^ *P* < 0.05 indicates a significant number of miRNAs altered within a pathway/function.

**Table 3:** mRNAs altered by at least twofold after 4 h H_2_O_2_ exposure in villous 3A trophoblasts.

Gene symbol	Gene description	Fold change^b^
**HBEGF** ^a^	Heparin-binding EGF-like growth factor	5.7637
ANGPT2	Angiopoietin 2	−2.4932
CCL2	Chemokine (C-C motif) ligand 2	−2.3834
CD40LG	CD40 ligand	−2.2084
CP	Ceruloplasmin (ferroxidase)	−2.2705
CRH	Corticotropin releasing hormone	−2.1038
DCN	Decorin	−2.0042
HP	Haptoglobin	−2.4083
IGF1	Insulin-like growth factor 1 (somatomedin C)	−2.5991
IL18	Interleukin 18 (interferon-gamma-inducing factor)	−2.6445
IL1A	Interleukin 1, alpha	−2.0748
IL6	Interleukin 6 (interferon, beta 2)	−2.0181
LEP	Leptin	−8.4152
LPL	Lipoprotein lipase	−2.2238
MMP9	Matrix metallopeptidase 9 (gelatinase B, 92 kDa gelatinase, 92 kDa type IV collagenase)	−2.0748
NDRG1	N-myc downstream regulated 1	−2.5901
NTRK2	Neurotrophic tyrosine kinase, receptor, type 2	−2.6721
PAPPA2	Pappalysin 2	−2.3102
QPCT	Glutaminyl-peptide cyclotransferase	−2.5368
SERPINA3	Serpin peptidase inhibitor, clade A (alpha-1 antiproteinase, antitrypsin), member 3	−2.1705
TAC1	Tachykinin, precursor 1	−2.5368
TNF	Tumor necrosis factor	−5.1266

^a^Upregulated gene is in bold font.

^b^Fold change in H_2_O_2_ treated cells compared to nontreated control.

**Table 4:** Altered miRNAs with putative altered mRNAs targets after 4 h exposure to H_2_O_2_ in villous 3A trophoblasts.

miRNA name	Number of genes targeted by this miRNA	Number of target sites identified in target genes	Range of strength scores^a^	Target genes
hsa-miR-181a-5p	3	3	−0.4593 to −0.234	DCN, IL1A, TNF
hsa-miR-181c-5p	3	3	−0.4573 to −0.219	DCN, IL1A, TNF
hsa-miR-181b-5p	3	3	−0.4593 to −0.234	DCN, IL1A, TNF
hsa-miR-181d-5p	3	3	−0.4573 to −0.234	DCN, IL1A, TNF
hsa-miR-27b-3p	3	3	−0.203 to −0.0872	IGF1, LEP, LPL
hsa-miR-29a-3p	3	4	−0.4102 to −0.094	IGF1, LEP, LPL
hsa-miR-29c-3p	3	4	−0.4102 to −0.09	IGF1, LEP, LPL
hsa-miR-875-3p	3	4	−0.504 to −0.2443	CD40LG, NDRG1, TNF
hsa-miR-9-5p	3	3	−0.453 to −0.0467	IGF1, LEP, NDRG1
hsa-miR-454-3p	2	3	−0.3152 to −0.219	IGF1, TNF
hsa-miR-543	2	2	−0.39 to −0.259	ANGPT2, IL1A
hsa-miR-30e-5p	2	2	−0.452 to −0.1342	IGF1, IL1A
hsa-miR-30b-5p	2	2	−0.452 to −0.1236	IGF1, IL1A
hsa-miR-30a-5p	2	2	−0.452 to −0.1342	IGF1, IL1A
hsa-miR-30c-5p	2	2	−0.452 to −0.1236	IGF1, IL1A
hsa-miR-30d-5p	2	2	−0.452 to −0.1342	IGF1, IL1A
hsa-miR-424-5p	1	1	−0.0766 to −0.0766	IGF1
hsa-miR-152-3p	1	1	−0.3972 to −0.3972	IGF1
hsa-miR-148b-3p	1	1	−0.3972 to −0.3972	IGF1
hsa-miR-425-5p	1	1	−0.4961 to −0.4961	IGF1
hsa-miR-16-5p	1	1	−0.0977 to −0.0977	IGF1
hsa-miR-497-5p	1	1	−0.0766 to −0.0766	IGF1
hsa-miR-490-5p	1	1	−0.197 to −0.197	NTRK2
hsa-miR-193a-5p	1	1	−0.115 to −0.115	NTRK2
hsa-miR-151a-5p	1	1	−0.401 to −0.401	NTRK2
hsa-miR-330-3p	1	2	−0.05 to −0.01	NTRK2
hsa-miR-17-5p	1	2	−0.149 to −0.148	NTRK2
hsa-miR-1271-5p	1	1	−0.108 to −0.108	NDRG1
hsa-miR-20a-5p	1	2	−0.17 to −0.148	NTRK2
hsa-miR-154-5p	1	1	−0.263 to −0.263	NTRK2
hsa-miR-26b-5p	1	1	−0.1205 to −0.1205	IGF1
hsa-miR-766-3p	1	1	−0.161 to −0.161	IGF1
hsa-miR-190a-5p	1	1	−0.1862 to −0.1862	IGF1
hsa-miR-452-5p	1	1	−0.3111 to −0.3111	IGF1
hsa-miR-320a	1	1	−0.1466 to −0.1466	IGF1
hsa-miR-320b	1	1	−0.1466 to −0.1466	IGF1
hsa-miR-18b-5p	1	1	−0.2185 to −0.2185	IGF1
hsa-miR-374a-5p	1	1	−0.511 to −0.511	CCL2
hsa-miR-26a-5p	1	1	−0.105 to −0.105	IGF1
hsa-miR-222-3p	1	1	−0.1612 to −0.1612	IGF1
hsa-miR-221-3p	1	1	−0.1612 to −0.1612	IGF1
hsa-miR-192-5p	1	1	−0.2211 to −0.2211	IGF1
hsa-miR-196a-5p	1	1	−0.0267 to −0.0267	IGF1
hsa-let-7f-5p	1	1	−0.1633 to −0.1633	IGF1
hsa-let-7a-5p	1	1	−0.1592 to −0.1592	IGF1
hsa-miR-196b-5p	1	1	−0.0267 to −0.0267	IGF1
hsa-let-7b-5p	1	1	−0.1592 to −0.1592	IGF1
hsa-let-7i-5p	1	1	−0.1592 to −0.1592	IGF1
hsa-let-7e-5p	1	1	−0.1592 to −0.1592	IGF1
hsa-let-7d-5p	1	1	−0.1612 to −0.1612	IGF1
hsa-let-7c-5p	1	1	−0.1592 to −0.1592	IGF1
hsa-let-7g-5p	1	1	−0.1592 to −0.1592	IGF1
hsa-miR-576-5p	1	1	−0.1892 to −0.1892	IGF1

^a^Strength scores are the *Z* scores derived from the TargetScan algorithm. A more negative number indicates a stronger score and an increased likelihood that the gene is a bona fide target for the miRNA evaluated.
